# Evaluation of circumsporozoite protein of *Plasmodium vivax* to estimate its prevalence in the Republic of Korea: an observational study of incidence

**DOI:** 10.1186/1475-2875-12-448

**Published:** 2013-12-13

**Authors:** Pyo-Yun Cho, Sang-Wook Lee, Seong Kyu Ahn, Jin Su Kim, Seok Ho Cha, Byoung-Kuk Na, Yun-Kyu Park, Sung Keun Lee, Won-Ja Lee, Ho-Woo Nam, Sung-Jong Hong, Jhang Ho Pak, Yoon-Joong Kang, Youngjoo Sohn, Young-Yil Bahk, Han-Ik Cho, Tong-Soo Kim, Hyeong-Woo Lee

**Affiliations:** 1 Departments of Parasitology, Inha University School of Medicine, Incheon 400-712, Republic of Korea; 2 Department of Pathology, Immunology, & Laboratory Medicine, College of Medicine, University of Florida, J-566, 1275 Center Drive, Gainesville FL 32610, USA; 3 Department of Biomedical Technology, Inha University School of Medicine, Incheon 400-712, Republic of Korea; 4 Department of Parasitology and Institute of Health Sciences, Gyeongsang University School of Medicine, Jinju 660-751, Republic of Korea; 5 Department of Pharmacology, Inha University School of Medicine, Incheon 400-712, Republic of Korea; 6 Department of Parasitology, National Institute of Health, Osong 363-951, Republic of Korea; 7 Department of Parasitology and Catholic Institute of Parasitic Diseases, College of Medicine, Catholic University of Korea, Seoul 137-701, Republic of Korea; 8 Department of Medical Environmental Biology, College of Medicine, Chung-Ang University, Seoul 156-756, Republic of Korea; 9 Asan Institute for Life Sciences, University of Ulsan College of Medicine, Asan Medical Center, Seoul 138-736, Republic of Korea; 10 Department of Biomedical Science, Jungwon University, Goesan Chungbuk 367-805, Republic of Korea; 11 Department of Anatomy, College of Korean Medicine, Institute of Korean Medicine, Kyung Hee University, Seoul 130-701, Republic of Korea; 12 Department of Biotechnology, College of Biomedical and Health Sciences, Konkuk University, Chungju 380-701, Republic of Korea; 13 Korea Association of Health Promotion, Seoul 157-928, Republic of Korea

## Abstract

**Background:**

*Plasmodium vivax* re-emerged in 1993. Although the number of infections has been steadily decreasing, it is likely to continue to affect public health until it is eradicated. The aim of this study is to measure anti-circumsporozoite protein (CSP) antibody and compare malaria prevalence. As to understand the prevalence, an epidemiology study has to be conducted in the Republic of Korea.

**Methods:**

A total of 1,825 and 1,959 blood samples were collected in 2010 and 2011, respectively, from the inhabitants of Ganghwa and Cheorwon counties. The antibody titers of the inhabitants were measured by enzyme-linked immunosorbent assay (ELISA) using recombinant protein purified from *Escherichia coli* transformed with a CSP gene-inserted pET-28a(+) expression vector. Microscopic examination was performed to identify malaria parasites.

**Results:**

The annual parasite incidence (API) in Ganghwa decreased from 4.28 in 2010 to 2.23 in 2011, and that in Cheorwon decreased from 1.88 in 2010 to 1.15 in 2011. The antibody-positive CSP rate in these areas also decreased from 18.14% (331/1825) in 2010 to 15.36% (301/1959) in 2011. Pearson analysis showed a strong correlation between the API and the antibody-positive CSP rate in these areas (r = 1.000, *P* < 0.01). The intensity of the immune responses of the inhabitants of Cheorwon, as measured by the mean optical density, decreased from 0.9186 ± 0.0472 in 2010 to 0.7035 ± 0.0457 in 2011 (*P* = 0.034), but increased in Ganghwa from 0.7649 ± 0.0192 in 2010 to 0.8237 ± 0.1970 in 2011 (*P* = 0.006). The immune response increased according to age (r = 0.686, *P* = 0.041).

**Conclusions:**

The positive CSP-ELISA rate was closely related to the API in the study areas. This suggests that seroepidemiological studies based on CSP-ELISA may be helpful in estimating the malaria prevalence. Moreover, such studies can be used to establish and evaluate malaria control and eradication programmes in high-risk areas in Korea.

## Background

*Plasmodium vivax* is the causative agent of relapsing benign tertian human malaria, the second most common type of human malaria, and it afflicts several hundred million individuals annually. This disease is a major public health problem in most tropical regions and many temperate countries, including the Democratic People’s Republic of Korea (DPRK) and the Republic of Korea (ROK) [1].

The first scientific documentation of malaria occurrence was published in 1913, although malaria had been prevalent throughout the Korean peninsula for several centuries [2]. As a result of a national malaria eradication programme conducted in cooperation with the World Health Organization, the incidence of vivax malaria in ROK has rapidly decreased [3,4]. In fact, vivax malaria was thought to have been eradicated in ROK in the late 1970s until two sporadic cases were detected in the 1980s [5]. In 1993, one case was diagnosed in an ROK soldier serving in northern Gyeonggi province [6], and Cho *et al.* subsequently reported cases in two infected civilians [7]. Thereafter, many cases were reported near the demilitarized zone (DMZ), which centres on Paju-si, Yeoncheon-gun, Cheorwon-gun, Gimpo-si, Ganghwa-gun, Goyang-si, and Dongducheon-si. There is now considerable concern that malaria will become re-established in the region and then expand to other geographical areas [8].

Parasitaemia provides the basis for the classical method of measuring the endemicity of malaria. However, using data on only the incidence of parasitaemia may be insufficient to adequately determine the epidemiology of malaria in a given population. For example, when the incidence of malaria is low, mass blood surveys do not yield results commensurate with the work involved [9,10]. Serological surveys have provided valuable epidemiological information, especially in areas of low endemicity [11,12]. Interestingly, most patients with malaria in ROK show a long incubation period [13]. It is thought that a long incubation period is associated with sporogonic parasites; thus, circumsporozoite protein (CSP) was selected for the present seroepidemiological study. CSP is a sporogonic antigen and surface membrane protein that is expressed in all Plasmodium sporozoites. CSP has a central immunodominant region comprising a short tandem repeat of amino acid sequences containing multiple copies of the immunodominant B-cell epitope [14]. CSP is classified into two serotypes, VK210 [the dominant form, with GDRA(D/A)GQPA repeats] and VK247 [the variant form, with ANGA(G/D)(N/D)QPG repeats], which have different sequences in the repeated region of the CSP gene. It is known that the vivax malaria prevalent in Korea is the VK210 type [15].

**Figure 1:**
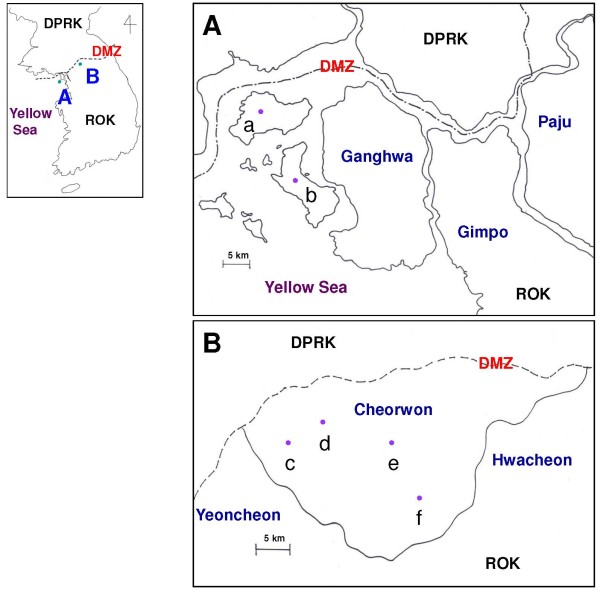
**Study areas. A**, Ganghwa county; **B**, Cheorwon county; a, Gyodongmyeon; b, Samsanmyeon; c, Cheorwoneup; d, Dongsongeup; e, Gimhwaeup; f, Seomyeon.

**Figure 2:**
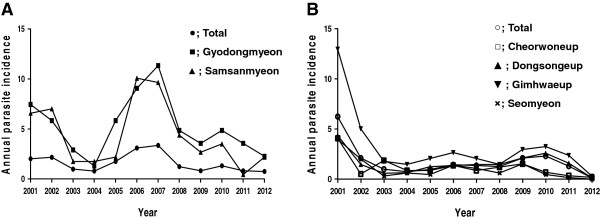
**Annual parasite incidence of study areas from 2001 to 2012. A**, Ganghwa county; **B**, Cheorwon county.

The annual parasite incidence (API) in Ganghwa county (Figure 1A) fluctuated from 2001 to 2012 (from 0.76 to 3.36) and peaked in 2007 (Figure 2A). Two islands, Gyodongmyeon and Samsanmyeon, which contain 24.5% (15.1%–30.9%) of patients with malaria among all patients in Ganghwa with malaria, were selected for the seroepidemiological study to evaluate the CSP antigen. In Cheorwon (Figure 1B), four administrative areas were selected for blood collection. The API of Cheorwon also fluctuated during the same time period (from 0.12 to 4.07) and peaked in 2001 (Figure 2B). The number of patients with malaria was 9.9% (2.9%–22.5%) in Cheorwoneup, 33.5% (20.4%–44.7%) in Dongsongeup, 12.6% (8.1%–16.7%) in Gimhwaeup, and 7.1% (0.0%–12.7%) in Seomyeon among all patients with malaria in Cheorwon county (Unpublished data, Korea Center for Disease Control and Prevention).

In this study, the dormant form of CSP was expressed and purified from *Escherichia coli* to produce antigen for the detection of anti-*P. vivax* CSP antibody levels in the inhabitants of Ganghwa and Cheorwon counties to evaluate CSP antigen in comparing the local malaria prevalence.

## Methods

### Study area

The study was conducted in Ganghwa county of Incheon Metropolitan city and Cheorwon county of Gangwon province, ROK. Ganghwa county (37°31′-45 N’, 125°33′-126°2′E) contains 179 villages (administrative village, -ri) in an area of 411 km^2^ that is covered with forested hilly mountains (181 km^2^, 44%), agricultural land (164 km^2^, 40%), building site (13 km^2^, 3%), and others (52 km^2^, 13%). The total population of Ganghwa county was 67,668 persons (male, 33,725; female, 38,943) living in 29,055 households in 2010 [16]. Cheorwon county (38°05′-24 N’, 126°56′-127°24′E) contains 109 villages (administrative village, -ri) in an area of 889 km^2^ that is covered with forested hilly mountains (601 km^2^, 67%), agricultural land (209 km^2^, 24%), building site (8 km^2^, 1%), and others (71 km^2^, 8%). The total population of Ganghwa county was 49,463 persons (male, 25,820; female, 23,643) living in 20,316 households in 2010 [17].

### Blood sample collection

The study locations are shown on the map in Figure 1. All of the study areas were near the DMZ, which is located along the borderline of DPRK. Blood samples were collected from participants residing in 32 villages among two administrative areas in Ganghwa county of Gyeonggi province and in 10 villages among four administrative areas in Cheorwon county of Gangwon province. It was conducted in November and December of 2010 and in November and December of 2011 in two areas of ROK: Ganghwa county of Incheon city, which contains two islands, Gyodongmyeon and Samsanmyeon (Figure 1A), and Cheorwon county of Gangwon province, which contains 3 –eup and 1 –myeon, Cheorwoneup, Dongsongeup, Gimhwaeup, and Seomyeon which is connected to DPRK by land (Figure 1B). To evaluate CSP recombinant protein as antigen for serodiagnosis, a total of 1,825 (4.77%) and 1,959 (5.12%) blood samples were collected in 2010 and 2011 from who wanted be a volunteer, respectively, from 38,288 total inhabitants in the study areas.

Blood smears were prepared for microscopic examination. Sera were separated and stored at -20°C for antibody analysis. Informed consent was obtained from all individuals. All samples were collected using human protocols that were reviewed and approved by the Human Ethics Committee of Inha University

### Microscopic examination

Thin blood films were prepared to determine the infectivity of blood samples. The blood films were fixed with methanol and stained with Giemsa stain diluted with buffered water at pH 7.2 to emphasize the parasite inclusions in the red blood cells (RBCs). The fixed monolayer of RBCs in this procedure makes the morphological identification of the parasite to the species level much easier and provides greater specificity than that obtained with thick-film examination. Thin blood films are often preferred for routine estimation of parasitaemia because the organisms are easier to see and count with this method [18]. To estimate the densities of blood-stage parasites by microscopy, it was counted the number of asexual parasites observed relative to 200 white blood cells (WBCs) and then multiplied the parasite:WBC ratio by 8,000, that is, the assumed number of WBCs per microlitre of blood [19].

### Amplification of the CSP gene

To express the CSP gene, genomic DNA was extracted from the whole blood of a patient with malaria using a QIAamp Blood Kit (Qiagen, Hilden, Germany). Polymerase chain reaction (PCR) was performed using AccuPower PCR PreMix (Bioneer, Daejeon, Korea), 50 ng of purified genomic DNA, and 40 pmoles each of forward (F1; 5′-CAC GTA GGA CAA AGT GCT AGC CG-3′) and reverse primer (R1; 5-′ATG GAC TCC ATG CAG TGT AA-3′). The total volume was adjusted to 50 μl with distilled water. The thermocycler conditions were as follows: denaturation at 94°C for 5 min; 35 cycles of 30 s at 94°C, 30 s at 55°C, and 45 s at 72°C; and finally, incubation at 72°C for 5 min. All PCR products were analyzed on a 1% agarose gel, confirmed under a UV transilluminator, and purified with a NucleoSpin Extract Kit (Macherey-Nagel, Duren, Germany).

### DNA sequencing and analysis

To genotype the CSP gene of *P. vivax*, the PCR product of the CSP gene was ligated into a pGEM-T Easy Vector (Promega, Madison, WI, USA) and transformed into *E. coli* DH5α. The PCR product containing *E. coli* DH5α was selected on ampicillin-containing medium [20]. To confirm the transformants, gel electrophoresis was performed with *Eco*RI digestion products after the plasmid was prepared with a Qiagen plasmid isolation kit according to the protocol supplied by the manufacturer. The CSP gene sequence was determined using an ABI PRISM Dye Terminator Cycle Sequencing Ready Reaction Kit FS (Perkin Elmer, Cambridge, MA, USA) according to the manual supplied by the manufacturer. M13 reverse and M13 forward (-20) primers were used in the sequencing. Nucleotide and deduced amino acid sequences were analyzed using EditSeq and Clustal in the MegAlign program, a multiple alignment program in the DNASTAR package (DNASTAR, Madison, WI, USA). The internet-based BLAST search program of the National Center for Biotechnology Information was used to search protein databases.

### Construction of the CSP expression vector

To express the CSP gene in *E. coli* DH5α, a CSP gene fragment was amplified from a blood sample that was confirmed to be infected with the dormant type of *P. vivax* as described above with the exception of the addition of the Fex2 (5′-ggatccAAAAAGGATGGAAAGAAAG-3′) and Rex2 (5′-aagcttGACTTTTCATTTGGGGCA-3′) primers, which contain *Bam*HI and *Hin*dIII sites on their 5′ ends, respectively. The amplified PCR products were digested with *Bam*HI and *Hin*dIII, purified with a Qiagen gel extraction kit after being run on an agarose gel, and then integrated into the *Bam*HI and *Hin*dIII cleavage sites of the pET-28a(+) expression vector (Novagen, 5,369 bp). The resulting plasmid was subsequently used for the expression of a CSP-(His)_6_ fusion protein in *E. coli*. The transformants were confirmed by both gel electrophoresis of plasmid DNA after restriction enzyme digestion with *Bam*HI and *Hin*dIII and DNA sequencing.

### Expression and purification of recombinant CSP

Expression of the recombinant protein was induced in *E. coli* DH5α with isopropyl-1-thio-β-D-galactopyranoside (IPTG) [10]. The CSP-(His)_6_ fusion protein was purified using immobilized metal ion affinity chromatography [21]. The purification was performed under native conditions according to the supplier’s protocol (Novagen). Proteins were analyzed by sodium dodecyl sulfate polyacrylamide gel electrophoresis (SDS-PAGE) after each purification step.

### Western blot analysis

The recombinant CSP-(His)_6_ fusion protein was separated on a 12% SDS-PAGE gel and transferred to a nitrocellulose membrane. After the transfer, the membrane was cut into strips and blocked for nonspecific binding with 3% skim milk for 12 h at 4°C. The membrane was then washed three times for 10 min each with 0.15% Tween 20-PBS. The strips were allowed to react with sera from patients with malaria or from uninfected individuals (diluted 1:100, vol/vol) for 4 h; they were then washed using the procedure described above. The membrane was subsequently incubated with diluted peroxidase-conjugated goat anti-human IgG secondary antibody (1:1,000, v/v) (Sigma) for 3 h at room temperature. For colour development, a solution containing 0.2% diaminobenzidine and 0.02% H_2_O_2_/PBS was applied to each well [22,23].

### Enzyme-linked immunosorbent assay

Enzyme-linked immunosorbent assays (ELISAs) were used to determine whether the blood samples contained antibodies against the CSP VK210 of *P. vivax*. Briefly, the capture antigen solution (50 ml, 0.5 μg/ml) was placed on a 96-well plate (Corning, Lowell, MA, USA) and incubated for 12 h at room temperature. The wells were aspirated and filled with blocking buffer (1% BSA, 0.05% PBS-Tween 20) and incubated for 1 h at room temperature. After the wells were washed with 0.05% PBS-Tween 20 three times, human serum samples in blocking buffer at a dilution of 1:100 (vol/vol) were added to the wells. Four positive and four negative control serum samples were also added to each plate. After 2 h of incubation at room temperature, the plates were washed with 0.05% PBS-Tween 20 three times, and peroxidase-conjugated anti-human IgG (Sigma, 1:2,000, vol/vol) diluted in blocking buffer was then added. The plates were re-incubated for 1 h at room temperature. The reaction was stopped by washing the plates as described above. To develop the colour, 100 μl of 2.2′-azino-di-(3-ethyl-benzthiozoline-6-sulfonic acid) peroxidase substrate (Kirkegaard & Perry Laboratories, Gaithersburg, MD, USA) was added, and the plates were incubated for 30 min. Absorbance was measured at 405 nm, and the cut-off value for positivity was defined as the mean + 3 standard deviations of the negative control samples. Negative sera were collected from volunteers among the staff of the Korea National Institute of Health (KNIH).

### Calculation of the annual parasite incidence

The annual parasite incidence (API) was calculated as the number of malaria-positive patients per 1,000 inhabitants for each of the study sites using microscopy: API = (number of positive slides / total number of slides) × 1,000.

### Data analysis

The differences in the positive CSP rates between 2010 and 2011 were determined by a Mann–Whitney test. Data analyses were performed using GraphPad software (GraphPad Software Inc., La Jolla, CA, USA). Pearson’s correlation analysis was performed to examine the relationship between seropositivity and the API of *P. vivax* in a given year. The data were analyzed using SPSS software, version 17.0 (SPSS Inc., Chicago, IL, USA). A *P* value of <0.05 was considered statistically significant. The correlation sizes were interpreted as none (0.0–0.09), small (0.1–0.3), medium (0.3–0.5), or strong (0.5–1.0) [24].

## Results

### DNA sequence of the VK210 Korean isolate

**Figure 3:**
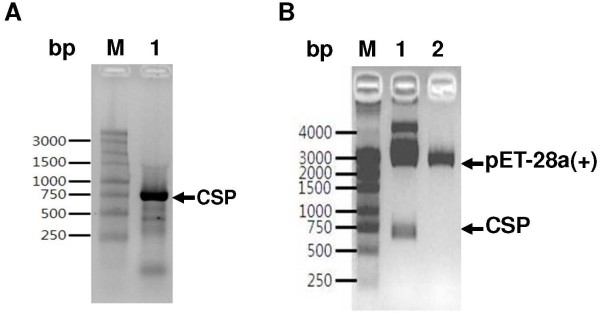
**Genetic cloning of circumsporozoite protein gene. (A)** Conformation of the PCR product of the circumsporozoite protein gene of *Plasmodium vivax* Korean isolate**.** M, Molecular size marker; lane 1, CSP gene. **(B)** Confirmation of circumsporozoite protein gene in *Escherichia coli* DH5α by restriction enzyme digestion with *Bam*HI and *Hin*dIII. M, Molecular size marker; lane 1; *Bam*HI and *Hin*dIII digested plasmid; lane 2, undigested plasmid.

The CSP gene that was amplified by PCR from genomic DNA was analysed on a 1.0% agarose gel. Amplification of the CSP gene yielded an approximately 750-bp DNA fragment (Figure 3A) that, after purification, was ligated into the pGEM-T Easy vector. The transformants were confirmed to contain PCR inserts by *Eco*RI digestion. The plasmid containing the PCR product was named pCS210 and was used for DNA sequence analysis. DNA sequencing revealed that the cloned CSP gene was 717 bp long and comprised 239 amino acids that were identified by DNASIS. The nonapeptide repeat unit, located between Region I (KLKOP) and Region II (PCSVT), showed the sequence GD(N)R(G)AD(G/A)GQP(A)A and was repeated 18 times.

### Expression and antigenicity of CSP VK210 type in *E. coli*

**Figure 4:**
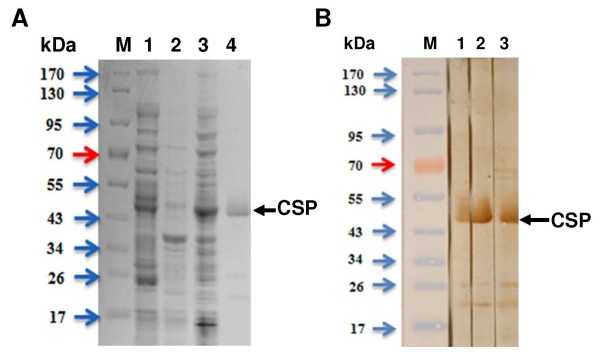
**Expression of circumsporozoite protein gene in *E. coli*. (A)** Purification of recombinant circumsporozoite protein with Ni-NTA agarose affinity chromatography**.** Lane M, Molecular weight protein marker; lane 1, induced *E. coli* DH5α cell lysate with IPTG; lane 2, flow-through; lane 3, wash; lane 4, elute. **(B)** Western blot analysis of recombinant circumsporozoite protein. M, Molecular weight protein marker; lanes 1–3, patients with malaria.

To generate the expression plasmid, the repeat region of the CSP gene was amplified from patient genomic DNA, digested with *Bam*HI and *Hin*dIII, and subcloned into the same restriction enzyme sites of expression vector pET-28a(+) (Figure 3B). The resultant plasmid, pCS210, contained the repeat region of the CSP gene fused to a (His)_6_-tag. The recombinant plasmid pCS210 was then transferred into *E. coli* DH5α. Next, 1 mM IPTG was added to cultures of *E. coli* DH5α (pCS210) grown to logarithmic phase in liquid LB plus 100-μg/ml ampicillin to induce expression of the target protein. SDS-PAGE followed by Coomassie blue staining showed that the molecular weight of the CSP recombinant protein was 45 kDa under native purification conditions (Figure 4A). The molecular weight of the target protein was twice as large as the expected molecular weight (24 kDa), which had been determined by DNASIS, it may affected by the repeated region of CSP.

**Figure 5:**
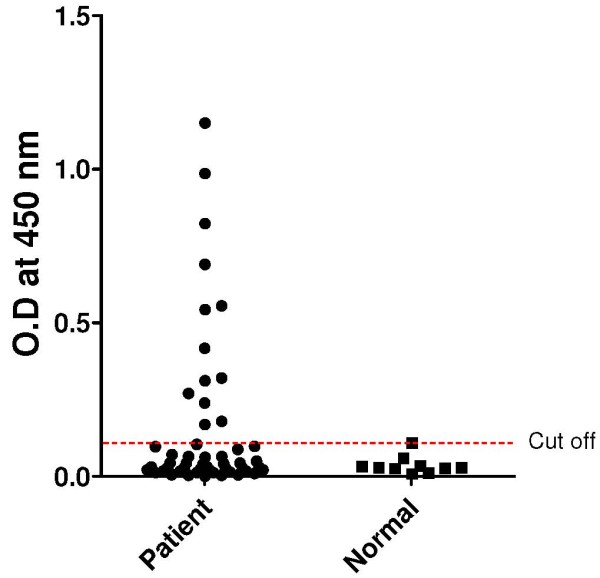
**Antigenicity of recombinant circumsporozoite protein.** Patient, individual infected with *Plasmodium vivax*; Normal, healthy volunteers.

The antigenicity of the CSP recombinant protein was determined by western blot. The sera of patients with malaria reacted positively (Figure 4B). To determine the sensitivity and specificity of the CSP recombinant protein by ELISA, the sera of patients with malaria, which had been reserved in KNIH after collection between 2009 and 2010, were used. Sera from 15 of 51 patients with malaria (sensitivity, 29.4%) were positive, while one serum sample from the normal control group (n = 10), who had never been exposed to malaria, was positive (specificity, 90.0%) (Figure 5).

### Local malaria transmission in Ganghwa

Two large islands located west of Ganghwa, Gyodongdo island (Gyodongmyeon) and Seokmodo island (Samsanmyeon), were also surveyed (Figure 1A). A total of 230 of 1,235 (18.62%) and 256 of 1,348 study subjects (18.99%) showed a positive response on CSP-ELISA in 2010 and 2011, respectively (Table 1). But there was no positive in microscopic examinations.

**Table 1:** Positive rates of circumsporozoite protein and annual parasite incidence in Ganghwa

**Area**	**No. of sera tested**		**No. of positive sera**		**Positive rate (%)**		**API**^**a**^	
	**2010**	**2011**	**2010**	**2011**	**2010**	**2011**	**2010**	**2011**
**Gyodongmyeon**	**933**	**966**	**196**	**179**	**21.01**	**18.53**	**4.86**	**3.56**
**Samsanmyeon**	**302**	**382**	**34**	**77**	**11.26**	**20.12**	**3.50**	**0.44**
**Total**	**1235**	**1348**	**230**	**256**	**18.62**	**18.99**	**4.28**	**2.23**

^a^API; Annual parasite incidence.

Gyodongmyeon displayed a higher positive rate in 2010 (21.01%, 196/933) than in 2011 (18.53%, 179/966). However, Samsanmyeon had a higher positive rate in 2011 (20.12%, 77/382) than in 2010 (11.26%, 34/302). The API for 2010 (4.28) was higher than that for 2011 (2.23). The APIs for 2010 (4.86) and 2011 (3.56) in Gyodongmyeon were higher than those for 2010 (3.50) and 2011 (0.44) in Samsanmyeon. The seropositivity of CSP in 2010 showed a strong positive linear relationship with the APIs of 2010 and 2011 (r = 1.000, *P* < 0.01). However, the seropositivity of CSP in 2011 showed a strong negative linear relationship with the APIs of 2010 and 2011 (r = 1.000, *P* < 0.01) (Table 1).

### Local malaria transmission in Cheorwon

A total of 101 of 590 (17.12%) and 45 of 611 (7.36%) study subjects in Cheorwon (Figure 1B) showed a positive response on CSP-ELISA in 2010 and 2011, respectively (Table 2). But there was no positive in microscopic examinations. Interestingly, Gimhwaeup exhibited a 50% (35/70) CSP-ELISA positive rate in 2010, but this rate dropped to 3.08% (2/65) in 2011. In 2010, the second highest positive rate (31.51%, 23/73) occurred in Seomyeon, adjacent to the eastside of Gimhwaeup; this location was ranked highest in 2011 (15.00%, 6/40). The third highest positive rate in both 2010 (10.85%, 28/258) and 2011 (6.08%, 20/329) was found in Dongsongeup, adjacent to the west side of Gimhwaeup. The lowest positive rate (7.94%, 15/189) occurred in Cheorwoneup, to the far west of Cheorwon, in 2010; however, this location had the second highest rate in 2011. In addition, Gimhwaeup showed the highest APIs in both 2010 and 2011 (3.24 and 2.36, respectively). These results suggest that Gimhwaeup, located in the centre of Cheorwon, was the focus of malaria transmission during the study period. The seropositivity of CSP in 2010 showed a moderately positive linear relationship with the API of 2010 (r = 0.435) and strong positive relationship with the API of 2011 (r = 0.509), but without statistical significance (*P* = 0.565, *P* = 0.491, respectively). However, the seropositivity of CSP in 2011 showed a strong negative linear relationship with the APIs of 2010 (r = -0.926) and 2011 (r = -0.931), but again without statistical significance (*P* = 0.071, *P* = 0.069, respectively) (Table 2).

**Table 2:** Positive rates of circumsporozoite protein and annual parasite incidence in Cheorwon

**Area**	**No. of sera tested**		**No. of positive sera**		**Positive rate (%)**		**API**^**a**^	
	**2010**	**2011**	**2010**	**2011**	**2010**	**2011**	**2010**	**2011**
Cheorwoneup	189	177	15	17	7.94	9.60	0.68	0.34
Dongsongeup	258	329	28	20	10.85	6.08	2.58	1.58
Gimhwaeup	70	65	35	2	50.00	3.08	3.24	2.36
Seomyeon	73	40	23	6	31.51	15.00	0.46	0.15
Total	590	611	101	45	17.12	7.36	1.88	1.15

^a^API; Annual parasite incidence.

### Intensity of immune responses

**Figure 6:**
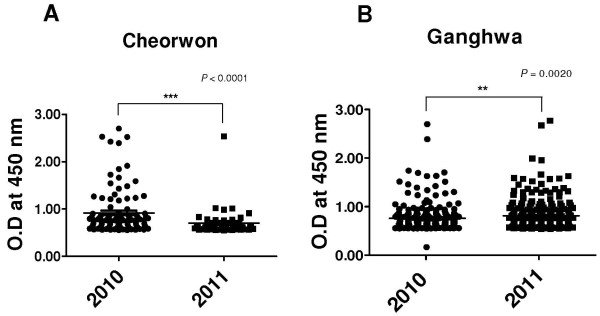
**Comparison of immune responses to CSP between 2010 and 2011.** Inhabitants of **(A)** Cheorwon and **(B)** Ganghwa.

The mean response intensity among 101 positive samples obtained from 590 inhabitants of Cheorwon in 2010 was 0.9186 ± 0.0472. This rate dropped to 0.7035 ± 0.0457 among 45 positive samples obtained from 611 inhabitants of Cheorwon in 2011 (*P* = 0.006) (Figure 6A). However, this rate did not change significantly in Ganghwa, increasingly slightly from 0.7649 ± 0.0192 among 230 positive samples obtained from 1,235 inhabitants in 2010 to 0.8237 ± 0.1970 among 256 positive samples obtained from 1,348 inhabitants in 2011 (*P* = 0.034) (Figure 6B). It should be noted that the study areas in Ganghwa were islands, and malaria transmission thus might not have been affected by foreign factors, whereas Cheorwon may have been affected by transmission from DPRK.

A total of 632 of 3,784 inhabitants exhibited a positive CSP response, and 625 of 632 positive cases could be categorized by age. Group A comprised CSP-positive inhabitants under 10 years of age who exhibited an immune response intensity of 0.7840 ± 0.0044. Group B comprised individuals between 11 and 20 years of age who exhibited an immune response intensity of 0.6974 ± 0.0435. Group C comprised individuals between 21 and 30 years of age who exhibited an immune response intensity of 0.7028 ± 0.0300. Group D comprised individuals between 31 and 40 years of age who exhibited an immune response intensity of 0.7730 ± 0.0375. Group E comprised individuals between 41 and 50 years of age who exhibited an immune response intensity of 0.7270 ± 0.0347. Group F comprised individuals between 51 and 60 years of age who exhibited an immune response intensity of 0.8272 ± 0.0379. Group G comprised individuals between 61 and 70 years of age who exhibited an immune response intensity of 0.7953 ± 0.0253. Group H comprised individuals between 71 and 80 years of age who exhibited an immune response intensity of 0.8435 ± 0.0264. Finally, Group I comprised individuals over 81 years of age who exhibited an immune response intensity of 0.8166 ± 0.0409. A strong positive correlation was shown between immune response and age (r = 0.686, *P* = 0.041) (Table 3).

**Table 3:** The immune responses to CSP in inhabitants according to age

**Group**	**Age**	**Optical density**	**Standard deviation**
A	<10	0.7840	0.0044
B	11-20	0.6974	0.0435
C	21-30	0.7028	0.0300
D	31-40	0.7730	0.0375
E	41-50	0.7270	0.0347
F	51-60	0.8272	0.0379
G	61-70	0.7953	0.0253
H	71-80	0.8435	0.0264
I	80>	0.8166	0.0409

## Discussion

Our malaria research team is interested in diagnosing vivax malaria based on antibody detection. Although microscopic examination is the gold standard method for malaria diagnosis worldwide, it has disadvantages, including the need for trained experts, its time-consuming nature, and its tendency to miss cases of low parasitaemia levels. However, antibody detection can compensate for the disadvantages of microscopic examination. If an antibody detection method is applied in the field using a rapid diagnostic test kit, the method can be used without rigorous training and the time required for diagnosis will be reduced. Furthermore, most patients with malaria have antibodies against malaria parasites, even those with low parasitaemia levels, and this method can be applied on a large seroepidemiologic scale. In this study, the CSP of the vivax malaria antigen was used to seroepidemiologically evaluate its usefulness in understanding malaria transmission. The survey areas, Ganghwa and Cheorwon, are two re-emerging malarial outbreak areas in South Korea, and both areas are located within 10 to 15 km of the southern DMZ [8]. The incidence of malaria peaks in August after the rainy season and declines to baseline by the end of October. Therefore, blood collection was carried out between November and December, when the active anopheline population was diminished. The DMZ is a 4-km-wide, 250-km-long corridor that extends across the middle part of the Korean peninsula. No civilians have been allowed to enter the DMZ for more than 50 years; therefore, natural ecosystems and biodiversity are highly conserved in the DMZ [25]. Outbreak areas have been expanding yearly both south and east of the DMZ. Outbreaks in these areas are believed to have originated from the northern part of the DMZ. The re-emergence of malaria is presumed to have originated not from the immigration of infected people from the north, but from mosquitoes infected with *P. vivax* that flew from the north because human passage through the DMZ is almost impossible (although there are some exceptions in the Gaeseong Industrial Zone). The corridor is heavily fortified on both sides of the buffer zones with land mines and barbed wire fences. Therefore, it is believed that these areas are exposed to mosquitoes. To estimate the prevalence of malaria exposure in these high-risk areas in Korea, CSP recombinant proteins were applied. It is a sporogony-stage protein that exists on the surface membrane of all plasmodium sporozoites. CSP has a central immunodominant region comprising short tandem repeat amino acid sequences that contain multiple copies of the immunodominant B-cell epitope [14]. Because it is highly immunogenic and can induce a protective response in sporozoite-immunized experimental animals and humans, CSP is being investigated as a candidate for a human malaria vaccine. The immunodominant B-cell epitopes of CSP from a large number of *P. falciparum* isolates of diverse geographical origins and of a smaller number of *P. vivax* isolates have been conserved within the species [26]. Interestingly, the lifespan of the CSP antibody in human beings is within 27 days in inhabitants of Thailand and is not boosted by additional exposure to CSP antigen, i.e., additional infection by anopheline mosquitoes [27]. These findings led us to consider patients with malaria with long incubation periods who usually show malaria onset the year following a five-month-long winter season without mosquitoes. Furthermore, the mean incubation period of *P. vivax* has been reported to be as long as 279 ± 41 days (range, 153–452 days) [28]. This finding suggests that patients with these long incubation periods could display either an absent or reduced antibody level against the CSP antigen. The percentages of patients with short and long incubation periods were 25% and 75%, respectively [8]. However, it is possible that the patients with short incubation periods and an onset within 1 month after exposure to the CSP antigen delivered via infective anopheline mosquitoes had an elevated anti-CSP antibody level. This is why the CSP antigen was selected among the many possible malaria antigens. The hypothesis suggested by our malaria team is that the API of the relevant year includes patients with short incubation periods who were infected in the relevant year and patients with long incubation periods who were infected in the previous year. Therefore, the positive anti-CSP antibody rates were compared with the APIs for the relevant and subsequent years. The positive CSP-ELISA rate in 2010 was related to the 2010 malaria prevalence in Ganghwa and Cheorwon. This rate might be influenced by prevalence in patients with short incubation periods. The positive CSP-ELISA rate in 2010 was significantly related to the 2011 malaria incidence in Ganghwa and Cheorwon. This finding suggests that the malaria situation in the subsequent year can be predicted by the relevant positive CSP rate, and a portion of this rate might influence the incidence in patients with long incubation periods. In other words, the positive CSP rate could be considered to be the inoculation rate of sporozoites by infective vector mosquitoes. If a person is bitten by infective mosquitoes, he or she will show a positive response on CSP ELISA. However, it is very difficult to calculate how many individuals become patients after having been bitten by infective mosquitoes. This number is highly dependent on individual immune competency. Unfortunately, it was failed to identify parasite-positive inhabitants by microscopic examination of 632 CSP antibody-positive inhabitants. In addition, none of the CSP-positive individuals in 2010 become patients in 2011. However, the positive CSP antibody rate apparently affected the outbreak in the following year. This observation may indicate that the antibody-positive CSP rate is closely related to the community response rather than the individual response. However, in a preliminary study, the use of a blood antigen to detect an antibody with an indirect fluorescent antibody test (IFAT) showed that 16.67% (4/24) of individuals with IFAT seropositivity among inhabitants of Ganghwa became patients with malaria in the following year (authors’ unpublished data). In another study performed in Gimpo in 1999, 16 of 125 individuals with IFAT seropositivity (12.80%) were also positive for malaria by PCR detection. Blood samples with an antibody titer of >1:256 had a high positive rate by PCR analysis [29]. Even if it is needed to obtain more data, the differences in PCR positive rates between CSP and IFAT among antibody-positive cases are attributable to different antigens, one from the sporogony stage (CSP) and the other from the schizogony stage (IFAT). These differences suggest that the stage-specific antigen and antibody detection methods should be matched. Therefore, it is better to use liver biopsy samples to detect parasites that are present in individuals with antibody-positive responses. Because the CSP antigen is a sporogony-stage antigen, it can be observed in the early developing stage of hypnozoites, which are liver-stage parasites. However, there are ethical and economic problems in obtaining the liver samples necessary to detect hypnozoites by PCR. Therefore, the positive responses on CSP antibody assays did not coincide with the PCR responses for antigen detection that used only blood samples.

To evaluate malaria transmission in a given geographical region, many factors, including temperature, mosquito density, vector capacity, climate, rainfall, and humidity, should be considered [30]. Parasitaemia provides a classical means of measuring malaria endemicity. However, patient incidence alone cannot provide a complete understanding of malaria prevalence because many factors affect the malaria prevalence in ROK, including the population density of mosquitoes, vectorial capacity, long- to short-incubation-patient ratio, symptomatic to asymptomatic patient ratio, differences in rainfall and temperature, and immunity of the community.

## Conclusions

Antibody detection using CSP-ELISA may provide useful information regarding malaria prevalence in certain areas and individuals. These serological methods are useful in identifying areas that require malaria control and evaluating the surveillance system in certain areas.

## Competing interests

The authors declare that they have no competing interests.

## Authors’ contributions

TSK, SHC, YS, HIC, BKN, and HWL conceived and designed the study and contributed to the execution of the research. HWL and TSK wrote the manuscript. YS, BKN, and WON performed the statistical analysis. SHC, JHP, WON, SJH, WJL, SKL, YKP, PYC, SKA, JSK, and YYB collected the blood samples. SHC, YJK, PYC, SJH, WJL, and JHP performed CSP-ELISA. SWL (Eastside High School) who has been working ay the University of Florida involved in expression of CSP recombitant protein and performed ELISA. HIC also provided most of research funding for this study. All authors have read and approved the final manuscript.
